# Auditory change detection in schizophrenia: sources of activity, related neuropsychological function and symptoms in patients with a first episode in adolescence, and patients 14 years after an adolescent illness-onset

**DOI:** 10.1186/1471-244X-6-7

**Published:** 2006-02-08

**Authors:** Robert D Oades, Nele Wild-Wall, Stephanie A Juran, Jan Sachsse, Ljubov B Oknina, Bernd Röpcke

**Affiliations:** 1 Biopsychology Group, University Clinic for Child and Adolescent Psychiatry, Virchowstr. 174, 45147 Essen, Germany; 2 Institute for Occupational Physiology, University of Dortmund, Ardeystr.67, 44139 Dortmund, Germany; 3 Institute of Higher Nervous Activity & Neurophysiology, Burdenco Neurosurgery Institute, Butlerova Str. 5a, Moscow, Russia

## Abstract

**Background:**

The event-related brain response mismatch negativity (MMN) registers changes in auditory stimulation with temporal lobe sources reflecting short-term echoic memory and frontal sources a deviance-induced switch in processing. Impairment, controversially present at the onset of schizophrenia, develops rapidly and can remain independent of clinical improvement. We examined the characteristics of the scalp-recorded MMN and related these to tests of short-term memory and set-shifting. We assessed whether the equivalent dipole sources are affected already at illness-onset in adolescence and how these features differ after a 14-year course following an adolescent onset. The strength, latency, orientation and location of frontal and temporal lobe sources of MMN activity early and late in the course of adolescent-onset schizophrenia are analysed and illustrated.

**Methods:**

MMN, a measure of auditory change-detection, was elicited by short deviant tones in a 3-tone oddball-presentation and recorded from 32 scalp electrodes. Four dipole sources were placed following hypothesis-led calculations using brain electrical source analysis on brain atlas and MR-images. A short neuropsychological test battery was administered. We compared 28 adolescent patients with a first episode of schizophrenia and 18 patients 14 years after diagnosis in adolescence with two age-matched control groups from the community (n = 22 and 18, respectively).

**Results:**

MMN peaked earlier in the younger than the older subjects. The amplitude was reduced in patients, especially the younger group, and was here associated with negative symptoms and slow set-shifting. In first-episode patients the temporal lobe sources were more ventral than in controls, while the left cingular and right inferior-mid frontal sources were more caudal. In the older patients the left temporal locus remained ventral (developmental stasis), the right temporal locus extended more antero-laterally (illness progression), and the right frontal source moved antero-laterally (normalised).

**Conclusion:**

From the start of the illness there were differences in the dipole-model between healthy and patient groups. Separate characteristics of the sources of the activity differences showed an improvement, stasis or deterioration with illness-duration. The precise nature of the changes in the sources of MMN activity and their relationship to selective information processing and storage depend on the specific psychopathology and heterogeneous course of the illness.

## Background

The detection of a change in ongoing ambient auditory stimulation is an important preliminary requirement for the conscious organisation of an adaptive response to a significant event. The unusual sound could be an unexpected tone in a well-known piece of music, or the telephone ringing during a conversation. The change is detected automatically, but the altered behaviour requires controlled information processing beyond detection. The brain's response on detecting deviance is registered by an event-related potential (ERP) called mismatch negativity (MMN). This is recorded by subtracting the ERPs after a series of similar stimuli from that elicited by the unexpected tone. The procedure requires no task, and is thus well-suited for study in patients with schizophrenia. But what parts of the brain generate MMN activity and what mechanisms are involved?

Sources of neuronal activity have been reported for the auditory cortices and the frontal lobe [1,2]. The frontal sources lie in the right inferior/mid-frontal and left anterior cingulate gyri [3,4]. This is consistent with functional imaging of the activity generated by dissonant tones in music [5]. The activity of these sources represents the registration of a change and the mechanism for switching to a new mode of information processing [6,7]. Sources in the superior temporal lobe represent the short-term sensory memory trace for the currently usual sound [8,9]. This sensory memory has many features in common with an auditory working memory [10]. Information in working memory is organised in the inferior frontal region [11] where activity closely covaries with that in the superior temporal areas in imaging studies of auditory memory (e.g. in same-different judgments [12,13]). Like the phonological loop in working memory [14], the auditory sensory trace can be reactivated for 11–15 seconds after a stimulus [15]. A monitoring function is widely attributed to both working memory and to the automatic process underlying MMN [16], for which a supervisory attention system [17] and a store are essential parts [18]. There is much evidence for impaired auditory [19] and non-verbal working memory in schizophrenia [20], but are both the memorial (temporal lobe) and switching (frontal lobe) components of MMN also impaired? If so, then an examination of the sources should show how the impairment is expressed. Associations with conventional neuropsychological indicators of working memory and switching were explored..

A reduced MMN is widely reported in patients with schizophrenia with or without antipsychotic medication [21-23]. In contrast, borderline or non-significant reductions are reported for depressed and bipolar patients [23,24], and MMN with a normal amplitude was recorded in obsessive compulsive disorders [25]. Significant [26] or nonsignificant [27] decreases described at onset may get more severe after a longer illness [24,27-29]. They are often more marked in patients with non-paranoid or negative symptoms [27,30] and without hallucinatory or delusory symptoms [31]. Such negative features (e.g. flat-affect, alogia, social withdrawal, avolition) correlate with the severity of working memory difficulties [32].

Initial studies of MMN sources with magnetoencephalography (MEG: [33,34]) and ERP and imaging techniques (e.g. [35,36]) suggest impaired left-sided locations and source strengths. But these have concentrated on the temporal rather than the frontal lobe. As suggested above, such temporal lobe changes would be expected if the reduced MMN amplitude reflects in part impaired superior temporal function in categorization [37], sound identification [38] and auditory working-memory processes [39]. But if the supervisory attention system is also impaired, the frontal sources of activity may be altered. Evidence from MMN topography [40,41] and source modelling [42] has implicated frontal generator impairments in schizophrenia, and that in the early stages of illness there may be some further deterioration of these impairments reflecting illness-progression [42].

In this current report two groups of patients were selected to examine the hypotheses of there being impaired frontal sources and a progressive deterioration with course in schizophrenia. Patients were either experiencing their first episode as adolescents, or had been diagnosed initially in adolescence 14 years before. (It has been suggested that an onset in adolescence can lead to a severer course of illness [43].) We sought to explain the topographic pattern of MMN activity with brain electrical source analysis locating dipoles bilaterally in the frontal and temporal lobes based on published models [3,42]. This procedure describes 4 features (locus, orientation, strength and latency) for each of the 4 dipoles that could differ between groups. We concentrate on the MMN generated by stimuli of different durations for which impairments are frequently reported, and may even be impaired in the patients' relatives [44], making it a candidate for an endophenotype of change detection processes.

## Methods

### Participants

The study protocol was reviewed and approved by both the board of the University of Essen Psychiatry Clinics and the Ethics Committee of the Faculty of Medicine according to the criteria of the Declaration of Helsinki (00-2-1357-Y). After the procedures had been described all subjects and care-givers gave written informed consent. A first-episode of DSM-IV schizophrenia was diagnosed in 28 adolescent inpatients (early-onset: EOS) on the basis of a clinical interview and hospital records first on the ward and then by research group personnel. This was confirmed 6 months later to exclude affective, schizoaffective and schizophreniform psychoses. Eighteen outpatients who had their first break as adolescents in our clinic on average 14.4y before (SD 3.5, range 8.1–19.7: S-14Y) were also recruited: 11 showed a partial remission (CGI 3–5), and 7 a chronic course or no remission (CGI 6–8). They had a mean of 4.6 hospitalizations (range 1–12). Of 34 patients contacted, 9 declined and 7 were too ill: participants did not differ on mean age, gender, illness-severity or social function [45]. Patients were excluded for other major psychiatric or somatic illness, alcohol abuse in the last 5 years and current substance abuse other than nicotine. Two age-matched comparison groups were recruited by advertisement and paid for participation (C-EOS, C-14Y). None had used drugs affecting the central nervous system or had a history of neurological or psychiatric illness. Gender balance was not considered significant as gender does not influence MMN [1,46-48]. All had normal or corrected to normal vision, and normal hearing on audiometric testing. In the S-14Y group 15 were receiving stable doses of antipsychotic medication. Of the EOS patients 15 had received the same treatment for >3 days, and 13 were examined without medication (Table 1). Assessment of the positive and negative symptoms in patients [49], SCID-II interviews and testing took place in the same week. Neuropsychological testing included a short IQ (4 WAIS sub-tests [50]), trail-making, digit-span forwards and backwards, logical memories, and visual reproduction.

**Table 1:** Characteristics of two groups of patients with schizophrenia and two groups of healthy comparison subjects (means and standard deviations)

	EOS (N = 28)		C-EOS (N = 22)		S-14Y (N = 18)		C-14Y(N = 18)	
	Mean	SD	Mean	SD	Mean	SD	Mean	SD
Gender m/f	21/7		12/10		12/6		7/11	
Mean Age (y)	17.5	(0.4)	17.6	(0.4)	32.1	(0.9)	30.4	(1.4)
Handedness*	27R, 1M		21R, 1L		16R, 1M, 1L		14R, 3M, (1 missing)	
Socio-economic status**	3.9	(0.4)	2.6	(0.2)	4.2	(0.4)	3.5	0.3
Short-IQ***	93.3	(4.0)	115.8	(4.2)	91.1	(4.1)	109.9	(4.0)
DSMIV diagnosis:								
Paranoid	22		-		8		-	
Disorganised	5		-		2		-	
Undifferentiated	1		-		1		-	
Residual	-		-		5		-	
Schizoaffective	-		-		2		-	
Ratings per question^+^								
SANS	2.13	(0.49)	-		1.68	(0.93)	-	
SAPS	1.10	(0.8.)	-		0.81	(0.75)	-	
Antipsychotic	478.1	(317.4)	-		366.0	(157.6)	-	
Medication (CPZ)^#^	-							
[*N*, Atypical/Mixed/Typical]	10/3/2		-		10/2/2		-	
[*N*, without Medication]	13		-		4		-	
Mean cigarettes/day/smoker	15	[14, 50%]	5	[20, 91%]	25	[7, 39%]	9	[16, 89%]
[Non-smokers: N, %])^##^								

*Edinburgh inventory [91](-100/-50 (left- [L]), -50/+50 (mixed- [M]), +50/+100 (right-handed [R]). ** Parent occupation, scale 1–6 [92] (EOS *vs*. C-EOS, t_40 _= 3.6, *P *< .01). ***Short-IQ (information, arithmetic, digit-symbol, block-design [50]: patients<age-matched controls (t_74 _= -5.5, *P *< .01). ^#^Chlorpromazine equivalents (CPZ: mg/day) for those on medication (t_26 _= -1.18, *P *= .25). ^## ^More cigarettes consumed by the EOS *vs*. C-EOS (t_46 _= 3.6, *P *= .001) and by the S-14Y *vs*. C-14Y groups (t_34 _= 3.6, *P *= .002). ^+ ^No group differences in the mean ratings/question for positive (SAPS: t_37 _= -1.2, *P *= .25) and negative symptoms (SANS: t_37 _= -1.9, *P *= .06).

### ERP measurements

An auditory oddball sequence (3 sinusoidal tones, 76 dBspl) was presented over 1600 trials. There was a pseudorandom sequence of standards (800 Hz, 80 ms, 10 ms rise/fall, p = 80%), frequency deviants (600 Hz, p = 10%) and duration deviants (40 ms, p = 10%) with each deviant preceded by at least one standard (stimulus onset asynchrony 850–1050 ms, mean 950 ms). During 4 blocks of 200 trials (passive auditory condition) subjects performed a simple visual red/green circle discrimination on a PC (50:50; subtending 3.8° at 1.5 m, changing at random every 1100 ms). Responses to the green target alternated between hands between blocks. Tone detection is not suppressed during various concurrent visual processes [51], nonetheless the stimulus onsets were controlled so as not to coincide. Four further audio-visual trial-blocks were presented with response to the frequency deviant (active auditory condition). This permitted an analysis of duration-deviant MMN in a state of attention to the auditory modality.

An EEG was recorded (Neuroscan, El Paso) from 31 tin electrodes (Fpz, Fz, Cz, Pz, Oz, FCz, CPz, F3, F7, F4, F8, FT7, FT8, C3, C4, P3, P4, CP3, CP4, T3, T4, T5, T6, TP7, TP8, M1, M2) in an electrode cap (Electro-Cap International: modified 10–20 system) with impedance <5 kΩ. A vertical EOG was recorded from the supra-orbital ridge of the right eye and a horizontal EOG from the outer canthus of the right and left eye to monitor blink-and eye-movements for rejection of artefacts (>50 μV) in EOG leads. Electrodes were referenced to linked-earlobes and the average-reference recomputed offline. A band-pass filter was set at 0.1–100 Hz. Data were digitised with 16-bit resolution, sampled at 500 Hz and stored on a hard disk. Records were epoched separately for each tone type with 100 ms prestimulus baseline and linear detrended. A 30 Hz low-pass filter (24 dB/octave) was used offline.

### ERP data analysis

**Figure 1:**
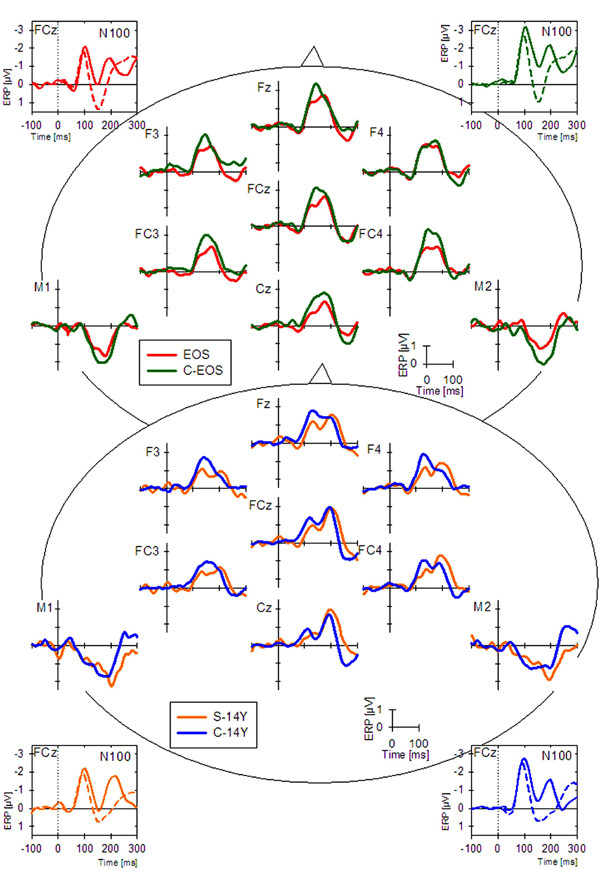
The central part of the figure illustrates the MMN waveforms recorded from 7 frontal and 2 mastoid electrodes for patients (EOS [N = 28], red; S-14Y [N = 18], orange) and age-matched controls (C-EOS [N = 22], green, C-14Y [N = 18], blue) during the passive auditory condition. The inserts show the waveforms elicited by the standard and the duration deviant tones recorded from FCz in the younger (top) and older subject groups (bottom). They illustrate the N1 peaks over 50 ms before the MMN peak. Nearly 50 ms after the MMN peak, in the deviant waveform, is a peak probably belonging to the N2/N2b family that contributes to the later part of the double-peak form in the subtraction-waveform.

The MMN waveform was derived by subtracting the ERP to standard tones from those elicited by the duration-deviant. Peaks were sought automatically from 90 to 225 ms after stimulus-onset. The MMN was averaged across blocks for each group and the mean amplitude was computed in successive 30 ms windows from average-refenced data (105–135–165–195–225 ms) for the active and passive condition, respectively. An initial repeated-measures analysis of variance (ANOVA) was carried out for 4 subject groups (between subject factor *group*) using data from 29 electrodes. The 135–165 and 165–195 ms windows covering the MMN peak (factor *window*), where inspection showed there to be potential group differences (Figure 1), were analysed by an ANOVA on the data from an array of 12 electrodes. The electrodes were arranged in two within-factors of 3 saggital chains (factor *side*: i.e. left; F3, FC3, C3, CP3: midline Fz, FCz, Cz, CPz: right; F4, FC4, C4, CP4)) and 4 coronal rows (factor *row*:frontal; fronto-central; central; centro-parietal). This reduced the degrees of freedom that were also adjusted with the Greenhouse-Geisser epsilon. The MMN in the active condition as well as the latency were analysed by ANOVAs using the same factors (for the latter excluding the factor *window*). Where significant main effects or interactions involved more than two factor levels, additional ANOVAs were carried out to test simple effects. The influence of antipsychotic medication was examined with ANOVA and Spearman correlations in the EOS group. Exploratory correlations were sought for 3 sets of MMN measures (frontal, mastoid and dipole-moments) with the CGI sum scores, the main clusters of SANS/SAPS symptoms, and neuropsychological measures of processes putatively related to MMN measures (digit-span, trail-making)[52,53]. Trends are described at *P *< .05 and type-1 corrected correlations at *P *< .002.

### MMN source analysis

**Figure 2:**
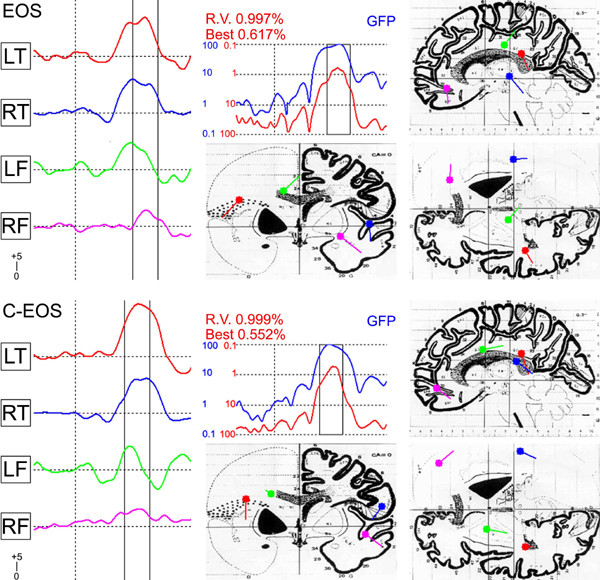
Principle components confirmed that bilateral frontal and temporal lobe dipoles were necessary to explain >98% of the topographic variance. The 4-dipole-model [3] was fitted to the ERP grand mean for each group. Each dipole was initially constrained to the hypothesis and then allowed to move within the model without constraint. Stereotaxic coordinates were calculated with the method of Garneron [57] and group solutions placed on the modified Montreal Neurological Institute atlas. The residual variance (RV) and best fit for the BESA-calculated group dipole solution (RV<2%) is plotted in the middle. On the left of the figure, for the young patients (EOS [N = 28]) and age-matched controls (C-EOS [N = 22]), is the time course for activity of the four MMN dipole moments whose location is marked in the left/right frontal (LF/RF) and left/right temporal lobes (LT/RT) on axial, coronal and sagittal sections of the brain atlas in the middle and on the right. To improve the accuracy of source-analysis headshape was controlled by digitizing electrode sites relative to the nasion, left and right periauricular skull-landmarks with ultrasound (Zebris, Munich).

Brain electrical source analysis (BESA) using a four-shell head-model [54] was used to compute dipoles based on the average-referenced ERP from 20 ms before to 40 ms after the MMN peak. Modelling requires iterative fitting of the dipole location and orientation in a spherical head-model until the difference between the recorded and the calculated surface data is minimised (least square fit [55]). Efficacy was enhanced by seeding with previously published solutions [3,42,56]. The goodness of fit is expressed by the residual variance (RV), not explained by the model. Final solutions showed an RV of <2% (Figure 2).

To calculate statistical differences between groups, the individual solutions were re-calculated for each subject. Waveforms were high- (1.5 Hz, 6 db/octave) and low-pass filtered (15 Hz, 12 db/octave). Data were discarded if a criterion of RV<4% was not reached. This reflects the decreased signal-to-noise ratio of an individual's waveform. Two C-EOS, 6 EOS, 3 C-14Y, and 5 S-14Y subjects were removed (Ns = 20, 23, 15, 13 respectively). Dipole-locations were compared between groups with a MANOVA including the 4-level factor dipole as well as the between-subject factor group. The x, y, and z-coordinates of each dipole [57] and the phi and theta orientation angles were compared with post-hoc F-Tests. Group differences of peak dipole-strength and latency were determined by univariate ANOVAs using a 2-level between-subject factor for the younger and older groups and a 4-level within-subject factor dipole (left/right, frontal/temporal lobe loci).

## Results

Demographic, clinical, performance and ERP measures are presented in Tables 1, 2, 3. There were no group differences for hit rates on the visual vigilance control task, although the S-14Y group responded slower than the controls (F_3,79 _= 5.8, *P *< .01). Slow reaction times were confirmed for S-14Y patients in the auditory task (F_3,79 _= 3.9, *P *< .02). Here both patient groups made fewer hits than their comparison groups (F_3,81 _= 10.8, *P *< .01: Table 2).

**Table 2:** Response time and accuracy (mean and standard deviation) on an adjunctive visual vigilance test and an auditory discrimination of frequency deviant tones in younger and older groups of patients with schizophrenia and age-matched healthy comparison subjects

	EOS (N = 28)		C-EOS(N = 22)		S-14Y (N = 18)		C-14Y (N = 18)	
	Mean	SD	Mean	SD	Mean	SD	Mean	SD
Visual vigilance	554	[162]	496	[125]	664	[211]	467	[82]
response time (ms)^#^								
Visual vigilance	92	[26]	97	[8]	98	[4]	100	[1]
task (% hits)*								
Auditory discrimination	446	[92]	409	[61]	478	[80]	401	[61]
response time(ms)^##^								
Auditory	72	[23]	90	[11]	61	[29]	92	[12]
discrimination (% hits)*								

Response latencies of the S-14Y group on the visual (t_33 _= 3.6, *P *< .01)^# ^and the auditory discrimination (t_32 _= 3.2, *P *< .01)^## ^were slower: both patient groups were less accurate than their respective controls (t_47,34 _= -3.7, -4.2, *P *< .01) on the auditory task (*).

**Table 3:** ERP Characteristics for the Four Subject Groups

	EOS (n = 28)		C-EOS (n = 22)		S-14Y (n = 18)		C-14Y (n = 18)	
	Mean	SD	Mean	SD	Mean	SD	Mean	SD
MMN trials accepted	64	10	68	9	65	13	63	10
MMN latency (at Fz, ms)	158^1^	30	153^1^	19	187^2^	26	164	32
MMN amplitude Fz, (135–165 ms)	-1.1^a^	0.9	-2.1	1.1	-0.9	1.0	-1.5^b^	0.9
Cz,	-0.7^a^	1.1	-1.6	0.9	-0.6	1.2	-0.6^b^	1.2
Right mastoid, μV	+0.9^a^	0.9	+1.8	1.1	+0.9	1.7	+1.2^b^	1.0

Patients with early-onset schizophrenia (EOS) and 14 years after initial diagnosis (S-14Y) and their age-matched controls (C-EOS, C-14Y) : A significant difference for MMN amplitudes (average-reference 135–165 ms) was found for 4 groups(F_3,82 _= 5.1, *P *= .003). The effect was accounted for by the comparison between young subjects (F_1,48 _= 15.2, *P *< .001) – with patient amplitudes reduced [a], and the comparison for young *vs*. older controls (F_1,38 _= 6.0, *P *= .019) with amplitudes smaller in the older subjects [b]. Peak MMN latencies were earlier in younger than older patients^1^, and later in S-14Y *vs*. C-14Y^2 ^(see text).

### Scalp recordings

Initial analyses with 29 electrodes showed group differences for MMN amplitude (F_84,2296 _= 3.5, *P *< .001, ε =.16) in the 135–165 ms window (Figure 1). A trend difference continued in the 165–195 ms window (p = 0.1). EOS patients had a smaller MMN than the young controls (F_28,1344 _= 6.6, *P *< .001, ε = .16). The reduction in the older patients *vs*. their controls attained trend significance (F_28,952 _= 1.9, *P *= .1, ε = .15), but it should be noted that the older controls had a smaller MMN than the younger controls (F_28,1064 _= 2.8, *P *< .02, ε = .18). The MMN amplitudes did not differ between patient groups, and thus provide no evidence for a deterioration nor an improvement between onset (EOS) and later stages of the illness (S-14Y).

The ANOVA using mean amplitudes in two consecutive time windows confirmed the fronto-centrally pronounced MMN topography (factor *row*: F_3,246 _= 56.8, *P *< .001). MMN amplitudes in the frontal and fronto-central rows did not differ but were larger than in the other rows (Fs_1,82 _> 4.8, *P*s < .03). Further MMN amplitudes were largest at the midline electrodes (factor *side*: F_2,164 _= 3.3, *P *= .043). However, the topography did vary with the time window (*window *× *side*: F_2,164 _= 7.8, *P *= .001). The MMN was broadly distributed in the 135–165 ms window (no significant differences between the midline, left and right side): but in the 165–195 ms window the midline MMN amplitude was larger than on the left or right side (Fs_1,82_>9.7, *Ps *< .003).

Most importantly, the 4 subject groups differed significantly from each other (F_3,82 _= 5.1, *P *= .003). Simple effects confirmed differences between the EOS group and their adolescent controls (F_1,48 _= 15.2, *P *< .001) as well as between the adolescent and the adult control groups (F_1,38 _= 6.0, *P *= .019). Effects were specific to time window and row (*window *× *row *× *group*: F_9,246 _= 2.6, *P *= .041). The group effect was pronounced in the 135–165 ms interval (*group*: F_3,82 _= 7.2, *P *< .001) and reduced to a trend in the 165–195 ms window (*group*: F_3,82 _= 2.4, *P *= .072). At 135–165 ms tests of simple effects revealed differences between EOS patients and their controls at all but the centro-parietal row (Fs_1,48_>13.5, *P*s < .002). Differences between the control groups were most pronounced at the frontal and fronto-central row (Fs_1,38_>7.5, *P*s < .011). When compared to their adult controls the S-14Y group showed a smaller MMN amplitude only at the frontal row (F_1,34 _= 4.8, *P *= .036).

ANOVA revealed significant differences in MMN peak latency between groups (F_3,82 _= 7.6, *P *< .001). Especially the S-14Y group showed an increased MMN latency, both, when compared to their adult controls (F_1,34 _= 18.7, *P *< .001) and to the younger patient group (F_1,44 _= 12.1, *P *= .001: Table 3).

### Medication

An ANOVA comparing MMN amplitudes in 15 EOS patients with and 13 without medication was not significant (F_8,192 _= 1.3, *P *= .27, ε = .23). MMN amplitude tended to decrease more in the second window (165–195 ms) with dose of antipsychotic medication (CPZ equivalents: Fz, r = +.58, *P *= .03, FCz, r = +.52, *P *= .06). In other words there was a modest tendency for medication to sharpen the shape of the MMN waveform around its peak latency.

### Dipole source analysis

The 60 ms of activity around the MMN peak was well explained by the 4-dipole model [3]. With minor adjustments of orientation and location, the group-solution model showed a RV<1.8% (best fit 0.9%) for each group (Table 4). The left temporal source was close to the border of the superior temporal gyrus with the insula and parietal cortex (BA 22, 29), and the right temporal source lay slightly more rostro-ventrally on the border of the medial and superior temporal gyri (BA 21, 22, 42). The left frontal source lay posteriorly in the anterior cingulate cortex (BA 24). The right frontal source was on the border of the inferior and mid-frontal gyri (BA 10, 44, 47: Figure 2).

**Table 4:** Location, strength, latency and orientation of modeled dipole sources calculated for patient groups with early onset schizophrenia (EOS), 14 years after onset (S-14Y) and their age-matched controls (C-EOS, C-14Y)

Subject Group	Interval (ms)	RV/Best-Fit (%)	Left/Right	Temporal/Frontal	Location			Strength (nAm)	Latency (ms)	Orientation	
					x	y	z			phi	theta
C-14Y (N = 18)	120–180	1.75/0.60	L	T (STG)	-46	-39	13	19.9	136	51	127
			R	T (S/MTG)	49	-32	-2	20.9	136	67	109
			L	F (ACG)	-11	-18	34	15.9	138	55	101
			R	F (I/MFG)	34	52	-3	8.6	168	57	84
S-14Y (N = 18)	166–226	1.40/0.88	L	T (STG)	-52	-29	3	12.6	206	40	131
			R	T (S/MTG)	61	-18	-3	13.8	184	59	140
			L	F (ACG)	-2	-11	42	10.9	192	57	101
			R	F (I/MFG)	32	49	-1	7.8	220	59	108
C-EOS (N = 22)	120–180	0.99/0.55	L	T (ITP)	-40	-32	24	24.2	154	54	129
			R	T (STG)	61	-26	17	16.1	174	66	115
			L	F (ACG)	-21	1	33	10.9	134	57	114
			R	F (IFG)	49	45	-2	8.1	158	58	114
EOS (N = 28)	140–200	0.99/0.62	L	T (ITP)	-45	-32	23	18.5	170	49	121
			R	T (STG)	52	-18	1	15.9	140	55	109
			L	F (ACG)	-12	-17	33	12.6	136	50	99
			R	F (I/MFG)	30	36	-4	7.7	174	51	112

ACG, cingulate gyrus; ITP, insula-temporo-parietal junction; I/MFG, inferior/mid-frontal gyrus; S/MTG, superior/medial temporal gyrus; STG, superior temporal gyrus

Source locations calculated from the individual solutions (similar to the group solutions) differed significantly between the groups (Wilks Λ = .16, F_12,22 _= 9.9, *P *< .001). Potentially reflecting post-adolescent maturation the left temporal source was more lateral (10 mm) and ventral (12 mm) in the older *vs*. the younger controls (Fs_1,33_>19.8, *P*s < .001), while on the right it was more medial (9 mm) and ventral (14 mm: Fs_1,33_>13.3, *P*s < .001). The frontal source in the older controls was more posterior in the left cingulate (12 mm), and on the right it was more anterior (10 mm) and medial (13 mm) *vs*. the younger controls (Fs_1,33_>26.2, *P*s < .001).

**Figure 3:**
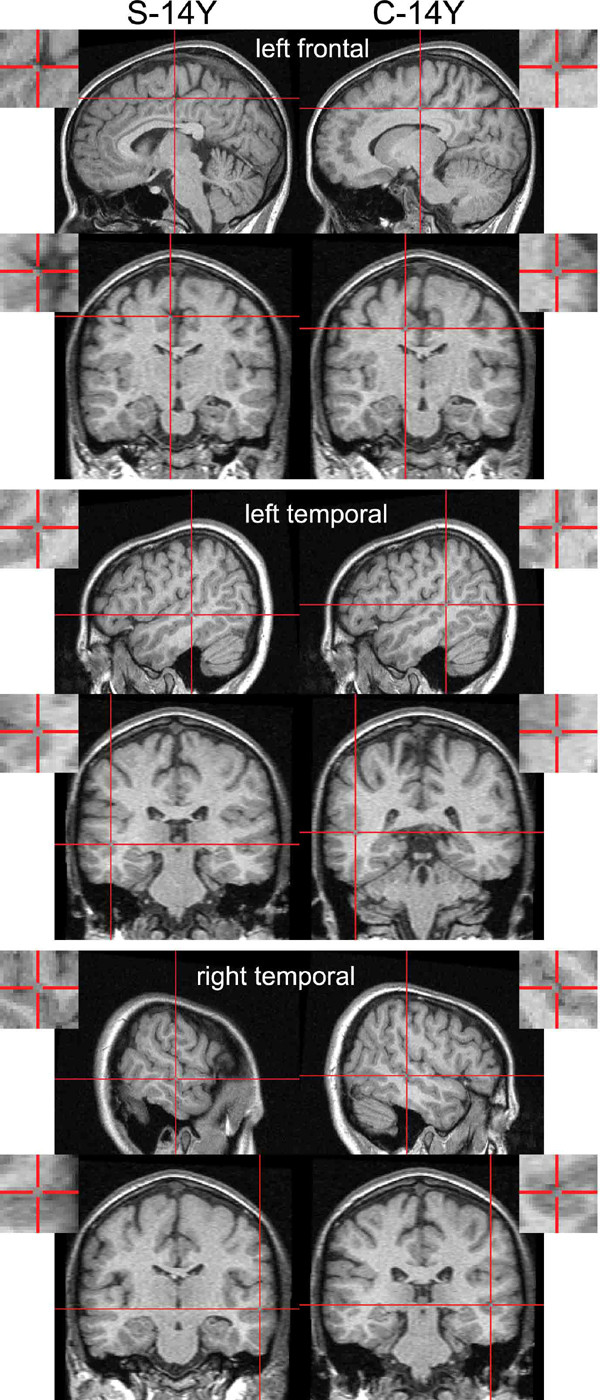
Group MMN dipole solutions for the older subjects (S-14Y [N = 18], C-14Y [N = 18]) are plotted for the left frontal (near cingulate), the left and right temporal lobe sources on sagittal and axial MRI scans of an individual brain from the C-14Y group (normalised on the SPM99 T1-brain template). Inserts show a close-up of the calculated source-locus. High resolution MR scans were obtained with T1-weighted 3D images, Siemens 1.5-T sonata: MP RAGE sequence, 384 × 384 × 192 matrix, resolution of 0.5 × 0.5 × 1.0 mm, repetition time/echo time/flip angle = 30 ms/10 ms/30° with a 256 mm field of view.

Compared to the young controls each dipole source in the EOS group was shifted slightly (Λ = .14, F_12,30 _= 15.4, *P *< .001). The left temporal source was more lateral (8 mm), and that on the right was 15 mm more ventral (Fs_1,41 _= 14.9, 48.0, *P *< .001). This is reminiscent of the adult control loci, but 11 mm more rostral. In the EOS patients the left cingulate source lay 15 mm more posterior, and the right frontal source 18 mm more medial than in the C-EOS group (F_1,41 _= 50.6, 73.5, *P *< .001: Figure 3). These values exceeded those in the same direction for healthy adults, and were less lateral (left cingular) and less rostral (right frontal).

**Figure 4:**
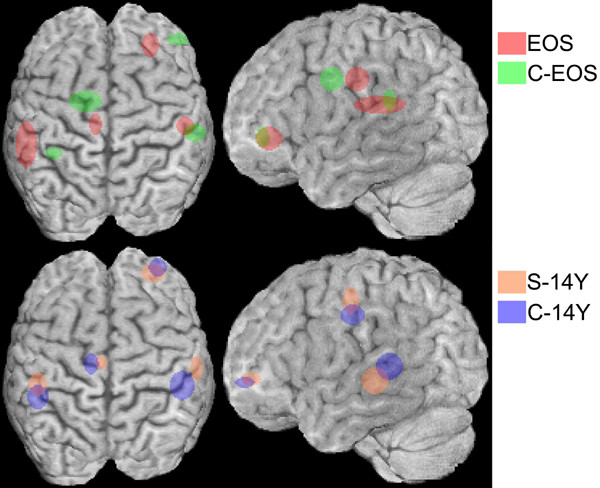
A view of the brain from above and the side with circles/ellipses showing the locus for the mean of the individual solutions and the spread of the individual source-solutions for 20 C-EOS, 23 EOS, 15 C-14Y and 13 S-14Y subjects. The radius in each dimension represents the standard deviation of the values for the constituent subjects.

Differences in the dipole locations in the S-14Y vs. C-14Y subjects were, like the MMN waveform, less marked than those in the younger groups (Λ = .25, F_12,15 _= 3.9, *P *= .008: Figure 4). The right temporal source was 10 mm more anterolateral and the left cingular source 7 mm more medial than in the older controls (F_1,26_>15.6, *P *< .001). These changes lay on different dimensions from those described between young patients and controls. Differences between older and younger patients that could reflect maturation or illness progression were evident (Λ = .14, F_12,23 _= 11.8, *P *< .001). The left temporal source was markedly more ventral (18 mm) and the right temporal source slightly more lateral (7 mm) in the older patients (F_1,34 _= 70.6, 15.1, *P *< .001). In contrast to the temporal source, the left frontal locus was 13 mm more dorsal and the right frontal locus 10 mm more rostral in the older than the younger patients (F_1,34 _= 26.6, 14.5, *P *< .001: Figure 3).

Dipole moments appeared weaker in patients (F_3,201 _= 4.4, *P *= .008). But ANOVA showed there were interactions with location rather than group. The right frontal source was always the weakest (*vs*. left cingulum; t_70 _= 3.1, *P *= .003). Likewise the slightly later dipole-latencies in patients were not significant (F_9,201 _= 1.2, *P *= 0.34). This is attributable to the variation recorded (SD 19–31 ms). However across groups mean latencies were longer for the right frontal source (173 *vs*. left frontal 161, left temporal 161, right temporal 163 ms: t_70_>2.8, *P *< .05) illustrating a bottom up sequence for activation. Finally although the dipole-orientation for the whole group solutions clearly differed between groups (see frontal and cingulate sources in Figure 2) comparisons of the means of the individual solutions proved variable, and ANOVA showed no significant group differences (see group data in Table 4).

### Active condition

Group differences similar to those in the passive condition, were found with attention to the auditory modality (F_3,77 _= 3.9, *P *= .012). An interaction of *group *× *window *indicated that a significant reduction of MMN amplitude in the EOS *vs*. C-EOS group was delayed to the 165–195 ms window (F_1,43 _= 5.2, *P *= .027). Differences for EOS *vs*. S-14Y (F_1,39 _= 7.1, *P *= .011) and C-EOS *vs*. C-15Y (F_1,38 _= 9.6, *P *= .004) were retained for the 135–165 ms window. But comparisons between the older subjects were non-significant.

Fitting the dipole-model from the passive to the active condition, resulted in a good fit for the young subjects (C-EOS, RV 2.1%, best fit 1.4%: EOS, RV 3.5%, best fit 2.1%). Peak-dipole activity in the older subjects also fitted well the 60 ms envelope around the peak (RV 5.1%, best fits 1.2–4.0%).

### Psychopathologic and neuropsychologic associations

Correlations were sought between MMN amplitude at Fz and the mastoids with the CGI and the 9 clusters of SANS/SAPS symptom ratings. EOS but not S-14Y patients showed decreasing amplitude with increasing CGI severity at frontal (r = +0.58, *P *= .002) and mastoid sites (r = -0.04, *P *= .035). In both patient groups associations of MMN with symptoms were restricted to 3 clusters. In EOS patients reduced MMN at the mastoids related to anergia (r = -0.42 to -0.7, *P *= .003–.0001) and flat affect (r = -0.43, *P *= .03). Quite separately in S-14Y patients there were indications that hallucinations related to decreased MMN at frontal sites (r = -0.54, *P *= .02), while, unexpectedly, increased amplitudes at the mastoids related to anergia ratings (r = +0.5 to +0.6, *P *= .03-.008).

Correlations for MMN amplitude with z-scores for trail-making measures of set-switching and digit-span measures of working memory were explored. Trails B-A scores were unrelated to MMN amplitude in healthy adolescents, but were negatively related to MMN amplitude at both mastoid sites in both time windows in EOS patients (r = -0.36 to -0.53, p = .07-.005). No correlations were evident in the older groups. There were no relationships to dipole-moments in the young subjects. But an association with the left cingular dipole in healthy adults (r = +0.67, *P *= .006) is of interest in view of trends in both older groups for an association of trails performance with temporal lobe dipole-moments in the left hemisphere (r = +0.5, *P *= .06–.08).

Surprisingly, in the C-EOS group digit-span scores correlated negatively with left and right mastoid MMN amplitudes in both time windows (r = -0.50 to-0.61, *P *= .01–.04). In contrast in EOS patients the correlations tended in the opposite direction at mastoid sites (r = +0.43, *P *= .03) and FCz (r = -0.4, *P *= .05). These measures were not correlated in the older subjects. There was no evidence of an association of MMN characteristics with other indicators of short-term memory (logical memories or verbal reproduction). Isolated relationships with dipole-moments could not be distinguished from chance.

## Discussion

Auditory change detection (MMN) was reduced in teenagers experiencing their first break of schizophrenia, compared to age-matched controls (mean 1.1 *vs*. 2.1 μV at Fz: Table 3). In outpatients, 14 years after their first episode in adolescence, the difference with young patients showed no clear signs of deterioration or improvement (mean 1.1 *vs*. 0.9 μV). The unexpected decrease of MMN amplitude in healthy adults compared to adolescent controls (from 2.1 to 1.5 μV) underlies the attenuated statistical difference between adult healthy and patient groups (see also [58]). The degree of reduction of MMN amplitude in the younger patients was similar to other reports, but the reduction in older patients compared to age-matched controls was slightly less than in other reports [21,23,44].

In all subjects MMN was calculated as reflecting bilateral source-activity in the temporal lobes and the left cingulate gyrus followed by the right inferior-mid frontal cortex in a bottom-up sequence. These source locations, originally described in Jemel et al. [3] have been broadly confirmed using independent components analysis, LORETA, fMRI and subdural recording [59]. Dipole strengths were decreased and their latencies delayed in the patient groups. But, significant illness-related changes in the strength of dipole activity could not be demonstrated due to the poor signal-to-noise ratio in the individual solutions derived for statistical testing for between group differences. However, source locations did vary significantly between groups (see sections below). These results, obtained during attention to a visual task, were well-replicated when subjects attended to the auditory modality. MMN expression was related to symptom severity, working memory and measures of set-switching (see below).

### On temporal lobe sources

In young first-episode patients sources in both the left and right auditory cortices appeared more to the left, and in the right hemisphere more ventral than in healthy teenagers. These are the same directions of change observed for the more caudally located sources in healthy 30- *vs*. healthy 17-year-olds. In these controls this likely reflects developmental white-matter expansion and grey-matter density increases [60,61] that can continue in the post-adolescent period [62]. The superficially similar result in EOS patients was unexpected. But this could reflect asymmetric ventricle expansion [63,64] that can be marked in early-onset patients [65].

In the older patients the left temporal source was more ventral than in controls or either group of young subjects. Indeed, their right temporal source changed in association with illness progression, being more anterolateral than in either the EOS or the C-14Y groups.

The reductions of MMN amplitude (significant), smaller dipole moments in the temporal lobe and slightly longer latencies (both non-significant) in adolescent patients are very similar to alterations reported for first-episode and adult patients [1,66]. There are reports of a loss of right MMN lateralization and reduced dipole-moments associated with location changes on the left [9,35,36]. Such changes are consistent with grey-matter loss in the left superior temporal gyrus, and specific to patients with a first episode of schizophrenia rather than other psychoses [67]. The location changes on the right, reported here, might be associated with the adolescent onset of illness. Grey matter reductions in the right superior temporal gyrus have indeed been reported for early-onset patients [68]. This would be consistent with earlier right hemisphere neurodevelopment [69], with the anomalies remaining 14 years after the adolescent onset.

### On frontal lobe sources

Frontal MMN sources varied more between groups on the rostrocaudal axis rather than on the lateral axis, as seen for sources in the temporal lobe. In the young patients the right frontal source was more caudo-medial than in their controls, but with similar lateral coordinates to the older controls. The dipoles were more rostrally located in younger and older controls. This more caudal locus in the patients is consistent with data for the frequency-deviant MMN [42] and was tentatively interpreted to reflect delayed development in that part of the forebrain which matures latest [60]. Indeed, prefrontal grey-matter is decreased specifically in adult first-episode patients with schizophrenia, rather than affective psychoses [70]. This could account for sources remaining in a caudal location. The right frontal source of the older patients was located similarly to that in the controls. Yet as this source lay more rostral to that in the EOS patients, it probably reflects normal developmental expansion in the third decade of life.

Possibly also reflecting the illness and its duration the left cingular source in the S-14Y group was more medial than the controls, and more dorsal than in the young patients. But like the younger patients the cingular source was more caudally located than in the controls. It is intriguing that the left cingular source in young patients was more medio-caudally located than in the young healthy controls, but in a similar locus to the older controls. This result has some similarity with the results for the other dipoles (above). But it differs from changes we reported for the detection of frequency-deviants [42], where the patients' dipoles were located rostral to the controls. Thus we re-examined the data with low resolution electromagnetic tomography (LORETA [71]). This confirmed a rather rostrally located bilateral activation of the medial frontal gyri, albeit at the expense of right frontal activity, precisely in the two analyses showing the most rostral BESA solutions (namely the frequency-deviant source in the older patients and the duration-deviant source in the controls). While the degree of blending of activated regions in the LORETA solution may be accounted for by the smoothing effect of the underlying algorhythm, it remains difficult to account for the different directions of source changes seen in patients with the two types of deviant stimulus.

### Associations of MMN

Studies of normal subjects demonstrate that temporal lobe contributions to MMN represent the short-term memory templates for standard tones against which deviants can be contrasted. Frontal sources, active after deviant detection, initiate a switch to controlled processing to allow the significance for response to be assessed [6,7]. The cingulate source, active simultaneously with those in the auditory cortices, may be involved in monitoring the ongoing situation prior to the later engagement of the inferior frontal regions when discord is detected. This is similar to the inferences from studies of the processing of incongruent stimuli [72]. Generically, the function is similar to the mismatch of expectations registered by the error-related negativity that also has BESA-calculated dipole-sources in the cingulate cortex [73].

Trail-making abilities to switch between sets are associated with prefrontal function [74]. Exploratory analyses indicated that in the more severely ill young patients a reduced MMN from the mastoids was associated with poor trail-making scores. This is taken as broadly supporting the attribution of the ability to switch between channels of information processing to one of the functions represented in MMN. (We note there is considerable reason to doubt whether MMN recorded at the mastoids uniquely reflects the output of supratemporal generators, discussion in [75].)

Imaging studies show that digit-span measures of working memory rely on recruiting activity in a network that includes the right dorsolateral prefrontal and anterior cingulate cortices [76]. Despite one negative report for healthy subjects [77] correlations with MMN were anticipated (see introduction). Superficially the correlations for improved digit-span measures with decreased mastoid measures of MMN in healthy subjects and vice versa in patients are the opposite of what would be expected if one predicts an association with MMN's mnemonic role. However, if the main function of deviant detection by MMN (at the mastoid) is the facility for switching (previous paragraph), then in the context of remembering a series of digits, longer spans with fewer errors would be more likely in those showing a less marked MMN. The association of longer spans with larger MMN in patients could reflect either the impaired switching facility or a relatively improved mnemonic role for MMN, perhaps reflecting the function of the supratemporal generator. This interpretation accords with the negative association reported for verbal memory performance with MR-activity in the right prefrontal region just posterior to the dipole source discussed here [78].

It is remarkable that the associations for SANS/SAPS symptom-clusters with frontal and mastoid MMN were largely restricted to negative symptoms of anergia and positive hallucinations. The former was associated with reduced mastoid MMN in the EOS group, as reported elsewhere for duration-deviant MMN in the right hemisphere [1]. In the S-14Y patients hallucination ratings were related negatively to measures at frontal electrodes, also reported previously for left hemisphere MMN recordings [36]. This is consistent with the attribution of these symptoms to impaired functional connectivity between the temporal and frontal lobes [79,80]. The prominent role for negative symptoms in the EOS group may reflect their prevalence and significance in the development of psychosis in adolescence, when the earlier onset has a poorer prognosis [81] and patients frequently show reduced frontal volumes [82]. However, we cannot exclude that this role would apply to chronic patients with prominent negative symptoms considering a report that MMN deficits in patients ill for 18 years accounted for 42% of the variance in general assessments of function [83]. Thus, here a positive association with MMN for the negative symptoms of anergia or apathy in the S-14Y group was unexpected. Speculatively this could be a sign of the sort of strategy (withdrawal) acquired by patients who have had to cope (relatively succesfully) with this illness for many years. This requires further investigation.

### The MMN model

Sources of MMN activity in the frontal, cingulate and temporal cortices have been proposed without detailed localization [1,2,84]. Details were restricted to MEG studies of the auditory cortices [33] until the report from Jemel et al. [3]. The reliability of such models depends on the signal-to-noise ratio. A reasonable effect size requires data for 6 electrodes per dipole from a thousand trials [85,86]. This was achieved here for the group solution (1174–1792 trials), where the stability (RV<2%) was assisted by being hypothesis led [3,42]. For the individual solutions, that provided a measure of variance between subjects, the data from 80% of the subjects still explained 96% of the variance in this model. Accuracy was facilitated by the digitization of recording sites with respect to individual skull markers. Further, we checked the stability of the group solution over successive millimetres away from the point solution [87] and found they were usually stable over 2–6 mm along each dimension. This order of magnitude for activated tissue is physiologically more plausible than the calculated point solution. Another estimate of the volume activated, or of the error in calculating its centroid is provided by the variability between subjects. Despite the poor signal-to-noise ratio this was usually well below a centimetre (Table 3). This upper limit for the model's accuracy matches that obtained from known modelled sources [88,89]. But, in addition to the problems of inter-individual stability of the model, error is introduced in the placing of sources on the brain atlas. Discrepancies between MRI anatomical representations of the locus of an individual's source and its localization on an averaged template can be marked. One study of this issue reported differences in nearly two thirds of the cases examined [90]. We have partly corrected for this by representing sources on the anatomy of one of our subjects (figure 4). But without constructing an average brain image from the participants, we have not accounted for the anatomical variability between our subjects.

## Conclusion

In summary, superimposed on the post-adolescent brain expansion manifest on the lateral axis in the temporal lobes and rostro-caudally in the frontal lobes, separate anomalies of structural-functional relationships develop along these 2 axes in psychotic patients. We propose that in the temporal lobes a ventro-lateral migration of the MMN sources occurs secondarily to ventricle expansion. In the frontal lobes the lack of a rostral migration of MMN sources reflects delayed post-adolescent development of prefrontal regions in psychotic patients. An MMN impairment was marked in association with symptoms of withdrawal (anergia, flat-affect) and a lack of cognitive flexibility (attentional set-switching).

Scalp records of MMN show a modest recovery of change-detection between the EOS and S-14Y groups. However, in both the younger and in the older patients dipole moments and latencies were diffusely weaker and delayed compared to controls. These results may reflect a modicum of adaptive plasticity. The significant movement of the centres of activity giving rise to the MMN dipoles may reflect this as plasticity, expansion or migration of these functional centres. Alternatively the changed locations may represent a progressive illness-dependent impairment requiring adaptation elsewhere in central nervous function.

## Abbreviations

BESA, Brain electrical source activity; CGI, Clinical global impression inventory; DSM, Diagnostic and statistical manual (of the American Psychiatric Association); EOS, Early-onset schizophrenia; ERP, Event-related potential; MEG, magnetoencephalography; MMN, Mismatch Negativity; MR(I), magnetic resonance (imaging); RV, Residual variance; SAPS/SANS. Scale for the assessment of positive/negative symptoms; SCID, Structured clinical interview for DSM schizophrenia; SD, Standard deviation;

## Competing interests

The author(s) declare that they have no competing interests.

## Authors' contributions

NWW carried out most of the recordings, and their analysis, and contributed to drafting the manuscript; SAJ assisted NWW with the recordings and contributed to the analysis. LBO carried out some early recordings and analyses. RDO conceived planned and organised the study, ran the initial recordings and drafted most of the MS. JS and BR were involved in recruitment, diagnosis and neuropsychological testing and initial analyses of these data. All authors read and approved the draft manuscript.

## Pre-publication history

The pre-publication history for this paper can be accessed here:


